# MicroRNAs Distinguish Cytogenetic Subgroups in Pediatric AML and Contribute to Complex Regulatory Networks in AML-Relevant Pathways

**DOI:** 10.1371/journal.pone.0056334

**Published:** 2013-02-13

**Authors:** Svenja Daschkey, Silja Röttgers, Anamika Giri, Jutta Bradtke, Andrea Teigler-Schlegel, Gunter Meister, Arndt Borkhardt, Pablo Landgraf

**Affiliations:** 1 Heinrich-Heine University Düsseldorf, Medical Faculty, Clinic for Pediatric Oncology, Hematology and Clinical Immunology, Düsseldorf, Germany; 2 Oncogenetic Laboratory, Department of Pediatric Hematology and Oncology, Justus-Liebig-University, Gießen, Germany; 3 Department of Biochemistry, Laboratory of RNA Biology, University of Regensburg, Regensburg, Germany; University of Barcelona, Spain

## Abstract

**Background:**

The role of microRNAs (miRNAs), important post-transcriptional regulators, in the pathogenesis of acute myeloid leukemia (AML) is just emerging and has been mainly studied in adults. First studies in children investigate single selected miRNAs, however, a comprehensive overview of miRNA expression and function in children and young adults is missing so far.

**Methodology/Principal Findings:**

We here globally identified differentially expressed miRNAs between AML subtypes in a survey of 102 children and adolescent. Pediatric samples with core-binding factor AML and promyelocytic leukemia could be distinguished from each other and from MLL-rearranged AML subtypes by differentially expressed miRNAs including miR-126, -146a, -181a/b, -100, and miR-125b. Subsequently, we established a newly devised immunoprecipitation assay followed by rapid microarray detection for the isolation of Argonaute proteins, the hallmark of miRNA targeting complexes, from cell line models resembling core-binding factor and promyelocytic leukemia. Applying this method, we were able to identify Ago-associated miRNAs and their targeted mRNAs.

**Conclusions/Significance:**

miRNAs as well as their mRNA-targets showed binding preferences for the different Argonaute proteins in a cell context-dependent manner. Bioinformatically-derived pathway analysis suggested a concerted action of all four Argonaute complexes in the regulation of AML-relevant pathways. For the first time, to our knowledge, a complete AML data set resulting from carefully devised biochemical isolation experiments and analysis of Ago-associated miRNAs and their target-mRNAs is now available.

## Introduction

AML in children is a clinically and genetically heterogeneous disease characterized by differentiation arrest and malignant proliferation of clonal myeloid precursors. It is the second most frequent hematologic malignancy, accounting for 15 to 20% of all childhood leukemia. The overall survival rate of pediatric AML patients has been increased from approximately 30 to 73%, however, nearly half of the pediatric patients relapse [1]. Therefore, risk-group classifications including prognostic markers as well as more targeted therapeutic approaches for treating pediatric AML are urgently needed. In adult AML patients, miRNAs can be used as biomarkers [2], and recently, first studies investigating the expression of selected miRNAs in 50 and 80 pediatric AML samples suggest the same for children [3], [4].

miRNAs are small (∼21 to 24 nt), non-coding, regulatory and highly conserved molecules found in humans, animals, plants and some viruses [5]. They regulate a variety of developmental and physiological processes like cell differentiation, apoptosis and immune responses [6] and their role in hematopoiesis is beginning to be appreciated [7]. Often, miRNAs are located in fragile sites or common breakpoint regions for chromosome aberrations that involve oncogenes or tumor suppressor genes in cancer cells [8]. Although around 70% of miRNAs are located in regions of leukemia-associated cytogenetic changes, only a subset (∼20%) of these miRNAs are expressed in a study surveying a panel of acute myeloid leukemia cell lines [9]. Loss of miR-145 and miR-146a results in a long-term myeloid disease in mice, and reintroduction of both miRNAs into AML cells significantly induced cell death and prevented growth *in vitro* [9]. Additionally, it was shown that expression profiles of miRNAs cannot only be used for distinction of leukemia of different lineages [10], but also for differentiation of cytogenetic subtypes of adult AML. Three independent studies demonstrate that the cytogenetic subtypes t(8;21), t(15;17) and inv(16) offer unique miRNA expression profiles [11]–[13]. It was shown that miR-126 is highly over expressed in t(8;21) and inv(16) and miR-224, miR-368 and miR-382 are exclusively over expressed in t(15;17) in adult AML patients [12]. Just recently, the expression of a single miRNA, miR-125b, was analyzed in 131 primary pediatric AML samples with also high expression in t(15;17)-positive patients [14]. Another study investigated four miRNAs, miR-29a, -155, -196a, and -196b, in 82 pediatric AML samples and observed higher expression of miR-196a/b and lower expression of miR-29a in MLL-rearranged pediatric AML, while this study was ongoing [4]. We here extend the previous pediatric AML studies by profiling 102 pediatric patient samples with a comprehensive and quantitative miRNA microarray approach.

miRNAs exert their regulatory function in cooperation with one of four Argonaute proteins (Ago) in humans, the core component of the RNA-induced silencing complex. In *D. melanogaster* as well as in *C. elegans* siRNAs and miRNAs are sorted into different Argonaute proteins [15]. Argonaute bound miRNAs are able to bind mRNAs and block their translation in a sequence and structure-dependent manner [16]. Several bioinformatic prediction algorithms were developed to predict miRNA-binding sites on mRNAs. However, the results of prediction methods hardly overlap [17] and hundreds of mRNAs for each miRNA are predicted, making it time and resource intense to experimentally confirm those in a comprehensive and unbiased fashion. Therefore, methods for biochemical isolation of the targeting complex are being devised recently. Differential binding of miRNAs to Argonaute proteins has not been investigated in detail in humans, however, in HEK293 cells and in Jurkat cells Ago1 and Ago2 as well as Ago2 and Ago3, respectively, bind to all miRNAs, albeit at different levels [18], [19]. In mouse skin Ago1-3 were just recently shown to bind highly similar miRNAs [20]. Thus, specific functions of the different human Ago proteins remain elusive [21]. Co-immunoprecipitation methods using Argonaute-specific antibodies for complex isolation without cross-linking prior to cell lysis are used followed by detection of associated RNAs via microarray technology [22], [23] or sequencing [24], [25]. In this study, we established a modified PAR-CLIP method [26] we termed PAR-CLIP-Array (Photoactivatable-Ribonucleoside-Enhanced Crosslinking, Immunoprecipitation, and Microarray Hybridization) including the use of monoclonal Argonaute antibodies and photo-activated UV crosslinking with 4′-thiouridine to enhance specificity of co-immunoprecipitation. For rapid detection of Ago-associated miRNAs and their target-mRNAs we used microarray technology and identified possible miRNA-mRNA binding sites using computational target prediction tools like TargetScan [27], PicTar [28] and miRanda [29].

We here present the first broad miRNA expression study of cytogenetically distinct pediatric AML samples from children and adolescents (under the age of 18) together with the comprehensive identification of mRNAs and miRNAs associated with the four different miRNA targeting complexes in two AML model cell lines resembling core-binding factor and promyelocytic leukemia. We bioinformatically deduced miRNA-regulatory networks that were enriched for previously identified AML-relevant pathways involving miRNAs differentially expressed between pediatric AML patients with translocation t(8;21) and t(15;17).

## Materials and Methods

### Ethics statement

Patient material was provided by the Children's University Hospital in Gießen under the direction of Prof. Dr. Jochen Harbott. All patient samples were obtained following informed written consent from legal guardians of the children. The samples were obtained in approved clinical studies of the German pediatric oncology and hematology society (GPOH) that were reviewed in appropriate ethical commissions. All personal data were encoded and obscured for privacy reasons.

### Patient samples, control samples and cell lines

102 pediatric AML patient samples were obtained at the time of diagnosis after informed consent from the Oncogenetic laboratory of the Pediatric Oncology and Hematology department of the University Hospital of Gießen (Table 1). In order to be able to statistically analyze the different cytogenetic subgroups, patient samples were positively selected for t(8;21), t(15;17), inv(16) and MLL-rearranged samples. This deviates from the general characteristics of the whole study cohort as referred to elsewhere [30]. Please note that the median white blood cell count of our cohort is higher than of the whole study cohort due to the enrichment of inv(16) and MLL-rearranged samples. Patients of these cytogenetic subgroups also have the highest white blood cell counts compared to the other cytogenetic subtypes of the whole study cohort [30]. The frequency of mutations in NPM1, CEBPA and FLT3, shown to be of prognostic importance especially in the normal cytogenetic subgroup, was not available for our patient cohort. 71 samples were obtained from bone marrow with a median blast content of 74% (range 20–100%), while 31 pediatric samples were obtained from blasts out of peripheral blood with a median blast content of 87% (range 50–100%). The blast content of bone marrow might be underestimated since only the peripheral blast count was available for some patients. Six adulthood AML samples with adequate RNA quality, three carrying translocation t(8;21) and three carrying translocation t(15;17), were obtained from the Munich Leukemia Laboratory at time of diagnosis following informed consent. Those were used to evaluate possible effects specific to our miRNA detection method when comparing differences in expression of the here reported childhood leukemia data to previously published data on adulthood acute myeloid leukemia. Cytogenetic characterization was performed routinely for diagnostic purposes on fresh material. All patient samples were stored at −80°C prior to RNA extraction. CD34+ cells were obtained from bone marrow of two healthy donors after informed consent, separated by using immunomagnetic bead separation (Miltenyi Biotec) and stored after separation in liquid nitrogen. AML cell lines KASUMI-1 (DSMZ Number ACC-220) and NB4 (DSMZ Number ACC-207) were obtained from the German collection of microorganisms and cell culture and were grown in RPMI 1640 (Gibco Invitrogen) with 10% (v/v) fetal bovine serum (PAA), 2 mM L-glutamine (Gibco, Invitrogen) and 100 U/ml penicillin/100 µg/ml streptomycin (Gibco, Invitrogen) under standard growth conditions.

**Table 1:** Clinical and cytogenetic characteristics of AML patients.

Characteristic	Pediatric AML cohort (n = 102)	Adult AML cohort (n = 6)
**Age, y**		
Median	10.3	44.8
Range	0.5–17.9	34.5–81.8
**Sex, no. (%)**		
Female	51 (50)	3 (50)
Male	51 (50)	3 (50)
**White blood cell count, ×10^3^/µl**		
Median	42.5	-
Range	0.9–440	-
**Bone marrow: blast content median % (range %)**	74 (20–100)	63 (60–65)
**no. of patients**	71	6
**Peripheral blood: blast content median % (range %)**	87 (50–100)	-
**no. of patients**	31	-
**Cytogenetic abnormalities, no. (% of patient cohort)**		
t(4;11)	1 (0.98)	-
t(6;11)	2 (1.96)	-
t(9;11)	16 (15.69)	-
t(10;11)	6 (5.88)	-
t(11;19)	5 (4.90)	-
other t(11q23)	3 (2.94)	-
t(15;17)	14 (13.73)	3 (50)
inv(16)	13 (12.75)	-
t(8;21)	24 (23.53)	3 (50)
normal karyotype	4 (3.92)	-
other	14 (13.73)	-
**French-American-British classification, no. (% of patient cohort)**		
M0	1 (0.98)	-
M1	4 (3.92)	-
M2	23 (22.55)	3 (50)
M3	12 (11.76)	1(16.67)
M4	11 (10.78)	-
M4eo	9 (8.82)	-
M5	25 (24.51)	-
M6	2 (1.96)	-
not determined	15 (14.71)	2 (33.33)

### Photoactivatable-ribonucleoside enhanced crosslinking and total cell extracts

Prior to UV-crosslinking 100–300×10^6^ cells were incubated with 100 µM 4′-thiouridine in growth medium in the dark for 14 hours. Subsequently, cells were washed with ice-cold PBS and irradiated on ice with 365 nm UV light (150 mJ/cm^2^). Afterwards, the cells were washed with PBS, collected by centrifugation at 500× g for 5 min, flash frozen in liquid nitrogen and stored at −80°C. Immediately preceding immunoprecipitation cells were lyzed in 3 volumes of lysis buffer (20 mM Tris-HCl, pH = 7.5; 150 mM KCl; 2 mM EDTA; 1 mM NaF; 0.5% Nonidet P-40; 0.5 mM DTT, EDTA-free protease inhibitor cocktail (Roche Diagnostics)) on ice.

### Antibody-bead binding and Immunoprecipitation of Argonaute proteins

40 µl of Sepharose Protein G beads (GE Healthcare) were washed twice with 1 ml lysis buffer. 600 µg total protein of Argonaute protein hybridoma supernatant [24] were added to the washed beads with lysis buffer to a final volume of 1 ml. Similarly, 60 µg rat IgG2a antibody (Abcam) was used as isotype control in parallel experiments. The antibody was bound to the beads rotating at 4°C overnight. 1 ml cell lysate was added to 40 µl prepared antibody-conjugated beads and incubated in a rotating wheel at 4°C for 4 hours. The immunoprecipitates were washed twice with 1 ml wash buffer (20 mM Tris-HCL, pH = 7.5; 0.05% Nonidet P-40; 5 mM MgCl_2_; EDTA-free protease inhibitor cocktail) containing 300 mM KCl, twice with wash buffer containing 500 mM KCl and once with 1 ml PBS and resuspended in 100 µl PBS. 20% of the immunoprecipitates were used for Western Blot analysis and the remaining 80% for RNA isolation.

### Protein gel electrophoresis and Western Blot

After SDS gel electrophoresis and transfer of proteins to HybondP membrane (GE Healthcare), membranes were blocked with 5% milk powder/0.1% Tween-20/TBST. Primary monoclonal antibodies against Ago1, Ago2, Ago3 and Ago4 diluted 1∶50 [24] and secondary polyclonal goat anti-rat antibody (Jackson Immuno Research) diluted 1∶10,000 were used for Argonaute protein detection.

### RNA extraction from Argonaute proteins and cDNA synthesis

Ago-associated RNAs were extracted according to the TRIzol protocol (Invitrogen). The RNA was precipitated with addition of 5 µg yeast tRNA and 3 volumes of 100% ethanol. Finally, the RNA was collected in 25 µl nuclease free water.

1 µg total RNA or 4.6 µl of Argonaute isolated RNA was used for cDNA synthesis. 9.7 µl of cDNA synthesis master mix (10 mM DTT, 1× SuperScript buffer, dNTPs 2 mM each, 0.33 µM OligodT primer, 0.33 µM Hexamer primer) was added and RNA was reverse transcribed using SuperScript III Reverse Transcriptase (Invitrogen). RNA was degraded with 20 µl of 150 mM KOH/20 mM Tris base 10 min at 90°C. The mix was neutralized with equimolar amount of HCl.

### Quantitative RT-PCR (qRT-PCR) and TaqMan miRNA assay

qRT-PCRs for mRNA measurements were performed using the SYBR Green PCR Master Mix (Applied Biosystems) on a 7900HT Fast Real-Time PCR System (Applied Biosystems).

The TaqMan miRNA Assays (Applied Biosystems) were used for detection of individual miRNAs using qRT-PCR according to the manufacturer's instructions (Applied Biosystems) with slight modifications. In brief, 10 ng of total RNA and 1.66 µl of Argonaute isolated RNA was used. The master mix for the cDNA synthesis contained 1 mM dNTPs, 3.3 U/µl MultiScribe Reverse Transcriptase, 1× Reverse Transcription buffer, 0.252 U/µl RNase Inhibitor, 20% TaqMan miRNA Primer and nuclease free water up to 5 µl total volume. Quantitative PCR was done as described by the manufacturer.

### miRNA microarray labeling, hybridization and analysis

For the microarray hybridization, a two-color miRXplore Microarray (Miltenyi Biotec) was used as previously described [31]. Briefly, 3 µg total RNA was labeled with Cy5-coupled pre-adenylated donors and the universal reference (pool of 493 synthetic human miRNA oligonucleotides, 1 fmol each) was labeled with Cy3-coupled pre-adenylated donors by ligation using the mutated and truncated RNA ligase Rnl2(1-249)K227Q. Position control oligos and calibration oligos (miRXplore Kit v8) were added to the labeling mix. Microarray hybridization was executed according to the manufacturer's instructions using the semi-automated hybridization machine aHyb (Miltenyi Biotec). Microarray scanning was performed on a GenePix Professional 4200 A microarray scanner (Axon Instruments, Molecular Devices).

Signal processing was performed using GenePix Pro 6 software (Axon Instruments, Molecular Devices). First, the average raw spot intensity values of each spot were subtracted from surrounding background signal. Spots possessing a background corrected signal intensity over 100 light units and over 50% of feature pixels with intensities more than two standard deviations above the background were applied for further analysis. Channel intensities for each color were corrected using the spiked-in calibration oligonucleotides. To allow multiple comparisons across microarrays the normalized color channel of the sample was normalized to the normalized color channel of the universal reference pool. Global normalization methods could not be applied, since the dynamic range differed substantially between samples and the reference. Additionally, synthetic spike-in control oligonucleotides were used as positive and negative controls. Data were submitted to Gene Expression Omnibus (GEO accession number GSE35320).

### Affymetrix-Chip hybridization of mRNAs

The labeling and preparation of Argonaute protein-associated mRNAs were executed according to the GeneChip 3′-IVT Express Kit User Manual (Affymetrix). The RNA was hybridized to GeneChip Human Genome U133A 2.0 Arrays (Affymetrix) using standard conditions.

Analysis of mRNA expression data was executed using the Robust Multichip Average algorithm (RMA) implemented in RMAExpress [32]. Probe expression values were background corrected, normalized and summarized to probe sets. Data were submitted to Gene Expression Omnibus (GEO accession number GSE35320).

### Bioinformatics and statistical analyses

Bioinformatics and statistical analyses were carried out with the R 2.11.1 software (R Project for Statistical Computing [33]) using log_2_-transformed ratio values. The Mann-Whitney-U test was used for identification of differentially expressed miRNAs of AML patient samples and the two-sided t-test for unequal variances was used for identification of Argonaute protein-associated miRNAs and mRNAs. Unsupervised hierarchical clustering was performed for both miRNA and mRNA microarray data. The package heatmap.2 was used to compute an enhanced heatmap. The dendrogram was produced by an agglomerative algorithm. The similarity of two elements was calculated by Euclidean distance and complete linkage. Fold changes between three biological replicates of Argonaute immunoprecipitates and three replicates of the isotype control experiment were calculated. Additionally, p-values were calculated using a two-sample t-test for unequal variances. miRNAs or mRNAs with a fold change of at least 1.8 for all three replicates and a p-value<0.05 were included in further analysis. The nearest shrunken centroid method implemented in the predictive analysis of microarray (PAM) package [34] was used to identify classifier miRNAs for the different cytogenetic AML subgroups and were tested using 1000 iterations of the implemented 10-fold crossvalidation method. miRNA binding sites on Argonaute-associated mRNAs were predicted with BioConductor 2.6 using the package RmiR and the function read.mir() [33]. Target predictions using Argonaute-associated miRNAs and mRNAs as defined above were performed using the databases TargetScan [27], PicTar [28] and miRanda [29]. miRNA and mRNA expression data were visualized in a graph using the software tool Exploratory Gene Association Networks (EGAN) [35]. miRNAs were summarized into sequence groups with high sequence similarities as previously described [31]. Argonaute-associated mRNAs were modeled on KEGG pathways together with their identified miRNA-binding sites. Enrichment of KEGG pathways associated to Ago-bound mRNAs was calculated on the whole set of over 42,500 human genes using a hypergeometric distribution model. Genes of Argonaute-associated mRNAs were grouped by functional term similarity using the “high” threshold settings of the DAVID algorithm [36]. Enrichment of functional annotations over the whole set of probed genes was calculated for each individual gene group and a common name was manually curated characterizing at least 70% of the respective gene group.

## Results

### miRNAs function as biomarker in pediatric AML

**Figure 1:**
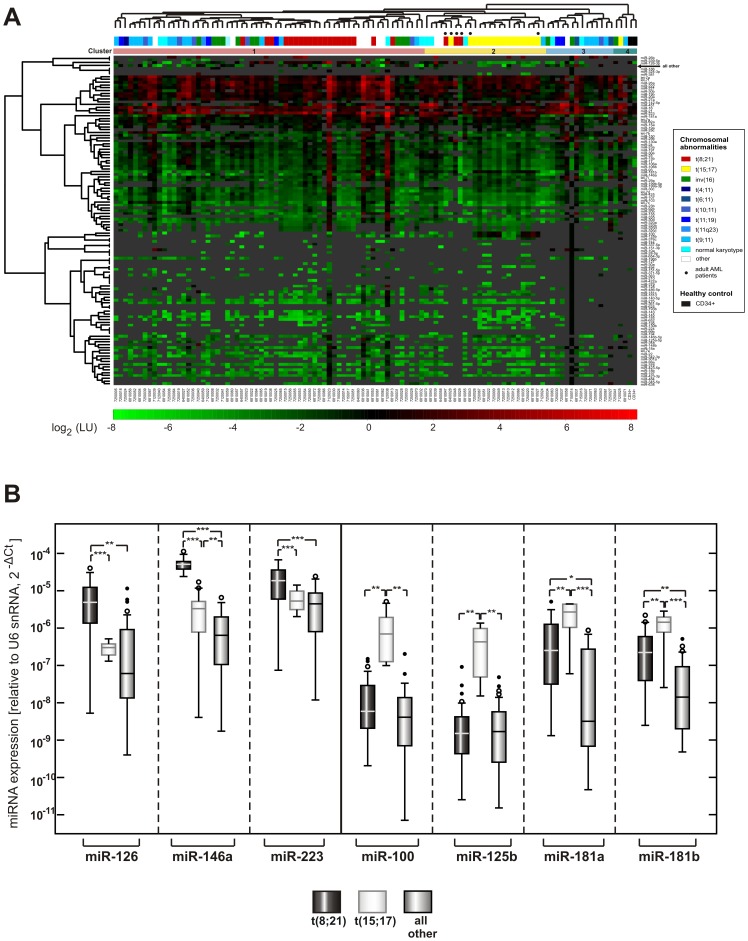
miRNA expression in pediatric and adult AML patient samples and healthy controls. (A) miRNA expression profiles of 102 pediatric AML patient samples of different cytogenetic aberrations as color-coded above the heat-map, six adult samples (•) and two CD34+ cell fraction from healthy donors were generated by a quantitative microarray approach. The heatmap represents miRNAs detected in at least 3 samples, while all others were summarized in “all others”. Background corrected signal intensities were corrected for each miRNA using 1 fmol of a universal reference consisting of synthetic ribooligonucleotides corresponding to 493 known human miRNAs. Unsupervised clustering of samples based upon the expression profiles of all miRNAs, indicated above the heatmap, reveals four distinct clusters with pediatric AML samples with translocation t(8;21) and t(15;17) clustering together and separate from each other. In contrast, adult AML samples cluster closely together irrespective of t(8;21) and t(15;17). (B) Validation of seven miRNAs most differentially expressed between t(8;21) AML samples and all other cytogenetic subgroups and t(15;17) APL samples and all other cytogenetic subgroups using TaqMan qRT-PCR. Expression values for t(8;21)-positive, t(15;17)-positive samples as well as cytogenetic subtypes other then the aforementioned ones are depicted. Please note that the selected miRNAs are also differentially expressed between these two cytogenetic subtypes. 22 patient samples with translocation t(8;21), 12 patient samples with translocation t(15;17) and 24 patient samples of all other cytogenetic subgroups were randomly chosen for validation. Indicated are the ΔC_T_-values relative to U6 snoRNA loading control. Expression differences were statistically analyzed using a Student's t-test as indicated; *p*<0.05 = *; *p*<0.01 = **; *p*<0.001 = ***.

We analyzed miRNA expression profiles of 102 pediatric AML patients in order to identify differentially expressed miRNAs discriminating between different pediatric AML subtypes (Table 1). We used a previously described and validated miRNA microarray platform [31], [37]. Unsupervised clustering divided patient samples into four major groups using solely miRNA expression data (Figure 1A). Pediatric AML samples carrying translocation t(15;17) and t(8;21) were completely separated from each other on the basis of their miRNA expression profiles, despite the great heterogeneity of all leukemia samples. In contrast, six adult AML patient samples with translocation t(8;21) and t(15;17) grouped together in a single cluster within Cluster 2 indicating a difference in their miRNA profiles, but mainly for the t(8;21) group. The MLL-rearranged samples were distributed in Cluster 1 and 3 together with inv(16). Independently, the nearest shrunken centroid method using the predictive analysis of microarray (PAM) algorithm on the whole dataset identified class predictors for t(8;21), t(15;17) and MLL rearranged AML patient sample, but not for the other cytogenetic subtypes. We identified a total of 15 miRNAs being significantly differentially expressed between MLL-rearranged and all other samples using the Mann-Whitney-U test (Table 2). Among them, the previously identified miR-196b [4] was expressed in 25 out of 33 MLL-rearranged patient samples with median expression strength of ∼30 pmol/µg total RNA, while this miRNA was expressed in only 12 out of 69 non-MLL-rearranged samples albeit at slightly higher expression levels (median expression level of ∼40 pmol/µg total RNA). Since we obtained 31 samples from blasts of peripheral blood specimen we analyzed the influence of blast origin on sample clustering. However, blast origin from either primary bone marrow or primary peripheral blood has no influence on clustering, possibly due to high blast content of the peripheral blood samples (median 87%; range 50–100%). Since translocation t(8;21) and t(15;17) were most distinct from each other in unsupervised hierarchical clustering, we identified differentially expressed miRNAs between these two groups and in comparison to all others (Table 2). miR-27a, -126, -150 and miR-223 were significantly higher expressed in t(8;21)-positive pediatric AML samples in comparison to t(15;17)-positive samples (Table 2). Conversely, miR-21, previously termed “onco-mir” due to frequent deregulation in several solid cancers, was significantly lower expressed in t(8;21)-positive samples in comparison to t(15;17) and all other cytogenetic subtypes. miR-100 was 10-fold more abundant in t(15;17)-positive leukemia than in any other cytogenetic subtype. Other differentially regulated miRNAs are given in Table 2. miR-126 and miR-100 were the most discriminating miRNA between t(8;21), t(15;17) and all other pediatric AML patient samples. We confirmed expression levels of selected miRNAs by quantitative real-time PCR (Figure 1B). 22 miRNAs differentially expressed between t(8;21), t(15;17) and MLL-rearranged AML samples were sufficient to correctly predict 62 out of 71 patient samples belonging to those three groups out of all AML patient samples (Table 2; Figure S1) while nine miRNAs (Table 2) were predictors when preselecting patient samples of these three cytogenetic subtypes. These are therefore candidate miRNAs for driving leukemogenesis in core-binding, promyelocytic and MLL-rearranged pediatric AML.

**Table 2:** miRNAs expression changes in translocation t(8;21)- and t(15;17)-positive as well as MLL- rearranged pediatric AML patients.

miRNA Name	t(8;21) vs. t(15;17)	t(8;21) vs. all other	t(15;17) vs. all other	MLL rearranged vs. all other
**let-7b^#^**	1.697 *	0.677	0.399 * ↓	0.980
**let-7c^#^**	1.427 *	0.757	0.530 * ↓	0.544 ↓
**miR-18b^#^**	1.846 ↑	0.762	0.413 * ↓	1.342
**miR-21^#^**	0.518 * ↓	0.634 *	1.224	9.818 * ↑
**miR-22^#^**	1.820 ↑	0.915	0.503 * ↓	1.093
**miR-24**	1.695	0.935	0.551 * ↓	1.147
**miR-26a^#^**	1.102	0.377 ↓	1.288	0.513 ** ↓
**miR-27a**	1.995 * ↑	1.029	0.516 * ↓	0.706
**miR-30c^#^**	1.133	1.288	1.137	0.461 * ↓
**miR-30d^#^**	1.787	1.218	0.681 *	0.464 * ↓
**miR-100^#§^**	0.056 ** ↓	0.672	11.986 * ↑	0.034 * ↓
**miR-125b^#§^**	0.157 ** ↓	1.332	8.496 * ↑	0.004 ** ↓
**miR-126-3p^#§^**	19.690 * ↑	4.313 ↑	0.219 * ↓	1.601×10^−4^ ** ↓
**miR-143^#§^**	1.635	1.316	0.805	0.031 ** ↓
**miR-146a^#§^**	1.592	3.223 * ↑	2.024 ↑	0.003 ** ↓
**miR-146b-5p^#§^**	0.707	1.673 ↑	2.367 * ↑	0.002 ** ↓
**miR-150^#^**	1.947 * ↑	1.039	0.534 * ↓	0.911
**miR-181a^#§^**	0.335 ↓	2.862 * ↑	8.550 * ↑	0.106 ** ↓
**miR-181b^#§^**	0.463 ↓	2.086 * ↑	4.501 * ↑	0.021 ** ↓
**miR-181d^#§^**	0.950	1.369	1.441	0.004 ** ↓
**miR-221^#^**	0.829	1.087	1.312	0.497 ** ↓
**miR-222^#^**	1.042	1.151	1.104	0.458 * ↓
**miR-223**	4.219 ** ↑	1.439	0.341 * ↓	1.767
**miR-342-3p^#^**	1.730	0.823	0.476 * ↓	1.656
**miR-378^#^**	1.901 ↑	0.897	0.472 * ↓	0.608

miRNAs distinguishing the two translocations from themselves or all other cytogenetic subtypes as well as miRNAs differentially expressed in MLL-rearranged samples compared to all other cytogenetic subtypes. Shown are those with at least 1.8-fold expression change (as indicated by the arrows) in a minimum of one comparison and fold changes of the comparisons are given. Mann-Whitney-U (MWU) statistical testing was performed for this comparison as indicated. Levels: *p*<0.05 = *; *p*<0.01 = **. miRNAs defined as class identifiers for the three cytogenetic aberrations using either patient samples of all cytogenetic subtypes (^#^) or the t(8;21)-, t(15;17)- or MLL-rearranged subtypes alone (^§^) using PAM are indicated as well.

Taken together, t(15;17)- and t(8;21)-positive pediatric patient samples could be distinguished from each other and from corresponding adult patient samples on the basis of their miRNA expression profiles in unsupervised hierarchical clustering in addition to the supervised prediction of MLL- rearranged patient samples based on the differential expression of just a few miRNAs.

### Global identification of miRNA and miRNA-targets in AML cell line models reveal varying binding preferences for different Argonaute proteins

**Figure 2:**
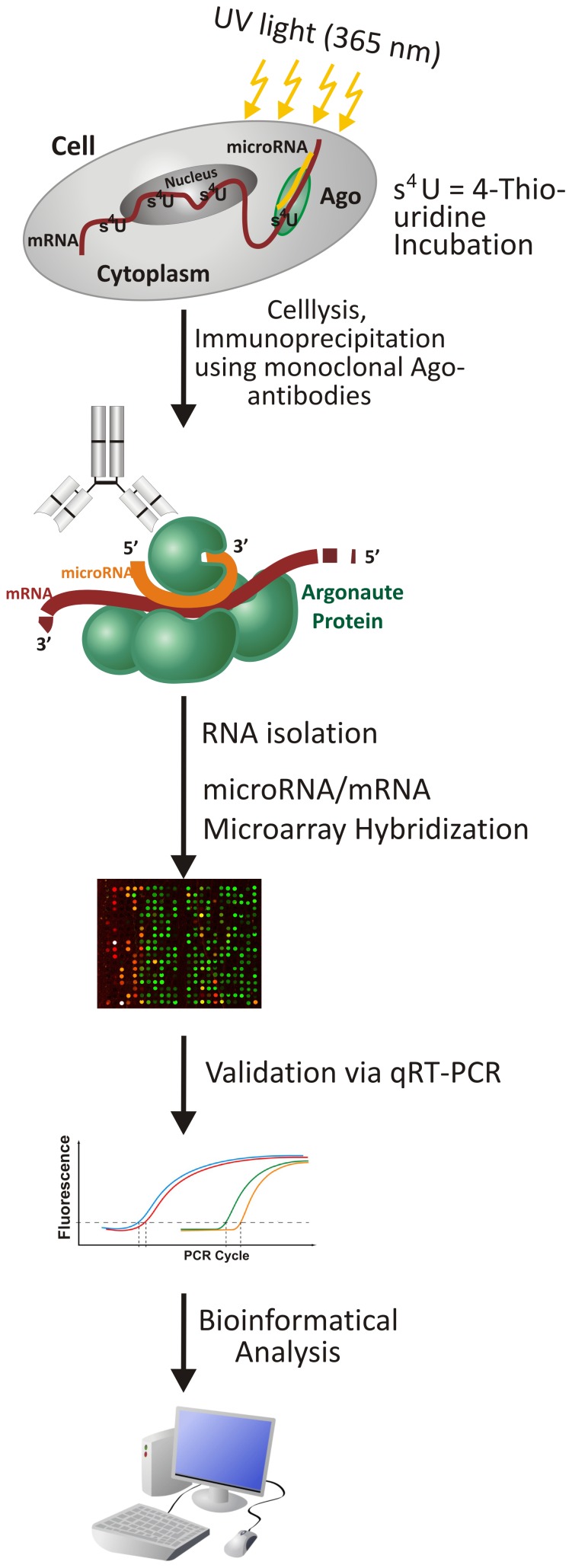
Schematic overview over the PAR-CLIP-Array method.

**Figure 3:**
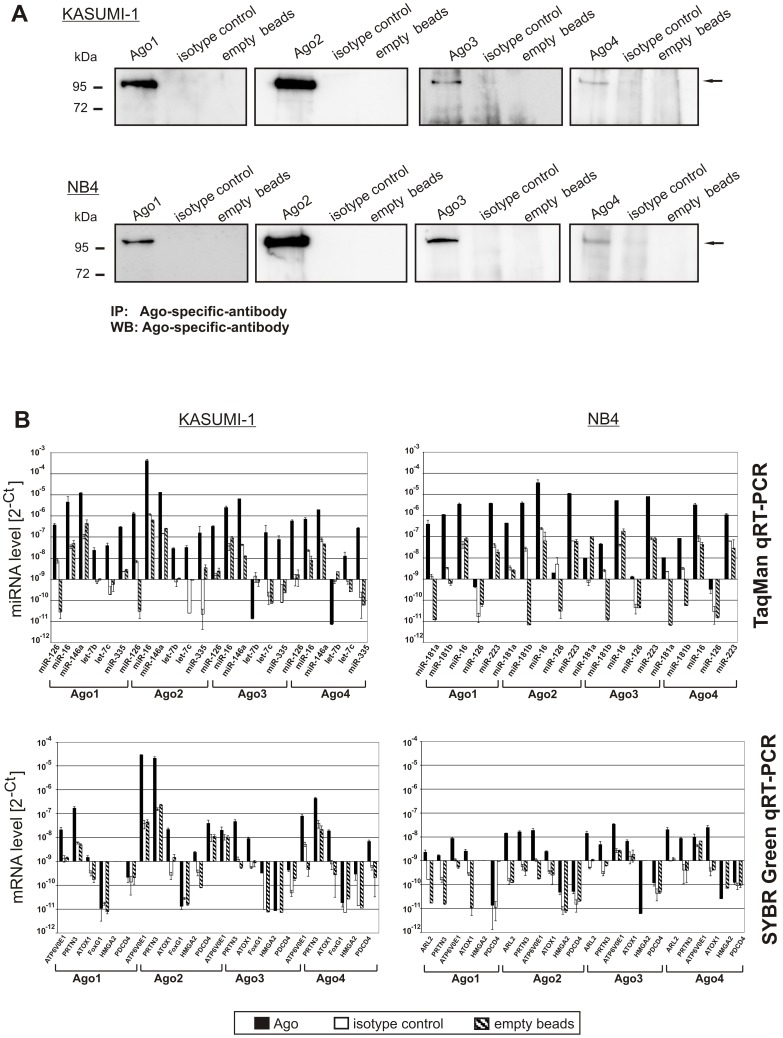
Ago-associated miRNAs and - mRNAs using the PAR-CLIP-Array method. (A) Western Blot analysis of immunoprecipitates of human Ago1-4 from AML cell lines, KASUMI-1 with t(8;21) and NB4 carrying t(15;17). The immunoprecipitates show a specific band of the Argonaute protein (∼97 kDa; ←) in contrast to the isotype control antibody (rat IgG2a) and empty-bead control. A representative sample of the biological triplicate is shown. Please note that more material was loaded for Ago3 and Ago4 since these two Ago proteins are much lower expressed as was also validated by qRT-PCR (not shown). Antibodies were tested for specificity for detection of native and denatured protein prior to this experiment with cell lines overexpressing tagged Ago protein (not shown) (B) Validation of miRNA- and mRNA-enrichment in immunoprecipitation experiments. Argonaute proteins (black bar) are compared to the isotype (white bar) and empty bead controls (grey bar) using TaqMan qRT-PCR assays for microRNA- (upper panel) and SYBR Green qRT-PCR assays for mRNA-quantification (lower panel). Shown are the measured levels (2^−C^T) of six and five miRNAs of KASUMI-1 cells (upper left panel) and NB4 cells (upper right panel), respectively. Immunoprecipitation experiments as well as cDNA synthesis were each done in triplicates and the mean value of the nine values as well as one standard deviation is depicted. miRNAs differentially expressed in patient samples between the t(8;21) and t(15;17) were selected together with ubiquitously expressed miR-16. Please note that calculation of ΔC_T_-values is not possible due to the lack of a housekeeping gene bound to Argonaute proteins. Six Ago-associated mRNAs in the KASUMI-1 cells (lower left panel) and NB4 cells (lower right panel), covering the whole range from low to high enrichment over isotype control according to microarray data, were selected for qRT-PCR validation. Graphs are centered around a C_T_-value of 29.9 cycles (2^−C^T = 0^−9^).

As miRNAs execute their regulatory function in the RNA-induced silencing complex containing one of the four human Argonaute proteins as core component, we biochemically isolated the Argonaute targeting complex and identified Ago-associated miRNAs and mRNAs. We chose the KASUMI-1 and NB4 cell lines because they also carry the translocations t(8;21) and t(15;17), respectively. Differentially expressed miRNAs at highest significance in t(8;21)- and t(15;17)-positive pediatric AML patient samples could also be detected in the total RNA of the respective cell line models (Table S1). We performed co-immunoprecipitation of all human Argonaute protein complexes with our modified PAR-CLIP-Array method using monoclonal antibodies, stringent washing conditions and photo-activated UV crosslinking with 4′-thiouridine in order to enhance specificity of co-immunoprecipitation and to avoid artificial resorting of bound RNA after cell lysis [38] (Figure 2). To account for unspecific binding to the beads and the Fc-part of the monoclonal antibody, we additionally used an appropriate isotype control rather than relying on a beads only- or non-immunized serum control. Ago3 and Ago4 protein precipitation yielded weaker bands than Ago1 and Ago2 (Figure 3A) in concordance with their expression levels as confirmed by qRT-PCR (not shown). Surprisingly, miRNAs as well as mRNAs associated more specific with different Argonaute proteins than expected from their structural similarities. Unsupervised hierarchical clustering separated the four Argonaute proteins from each other based upon the level of associated miRNA (Figure S2A) and, independently, mRNA (Figure S2B). Although differential binding of miRNAs to different Argonaute proteins has been reported for Ago1 and Ago2 in HEK293 cells [19] and for Ago2 and Ago3 in Jurkat cells [18], we identified miRNAs as well as mRNAs exclusively binding to one Argonaute protein, for the first time. This was especially pronounced in the myeloid leukemia cell line KASUMI-1 with 46 (48%) of all Ago-associated miRNAs specifically bound to Ago2, while 37% of miRNAs were associated with all four Argonaute proteins (Figure S3A). In contrast, in the more differentiated promyelocytic NB4 cell line only 8 miRNAs (9% of all Ago-associated miRNAs) were found solely in Ago2 complexes, while again about one third (31%) could be associated with all four Argonaute proteins in NB4 cells (Figure S3A). Only 89 mRNAs (8% of Ago-associated mRNAs) and 170 mRNAs (12% of Ago-associated mRNAs) were detected in all four human Argonaute proteins of KASUMI-1 and NB4 cells, respectively (Figure S3B). Remarkably, the hierarchical clustering of Ago-associated mRNAs showed also a complete separation of both cell lines indicating that each Argonaute protein works together with distinct subsets of miRNAs in order to regulate certain mRNAs differing between the KASUMI-1 AML cell line model with t(8;21) and the NB4 APL cell line model. Thus, Argonaute sorting is influenced by the cellular context through a yet undefined mechanism.

Subsequently, we used TaqMan and SYBR Green qRT-PCR for validation of the expression pattern of six and five Ago-associated miRNAs and six Ago-associated mRNAs of KASUMI-1 and NB4, respectively (Figure 3B). We chose miRNAs differentially expressed between t(8;21)- and t(15;17)-positive pediatric AML patients together with the ubiquitously expressed miR-16 and mRNAs over the whole expression spectrum as determined by our array method for validation. The expression was in agreement with both, array data generated from the Ago immunoprecipitates as well as data from our patient cohort. It also confirmed differential association of mRNAs to different Ago proteins in the two cell lines, again indicating an influence of the molecular miRNA sorting machinery on cellular context. For example, *ATP6V0E1* and *ATOX1* showed highest levels in Ago2 in KASUMI-1 cells, while in NB4 cells *ATP6V0E1* associated mostly with Ago3 and *ATOX1* mostly with Ago4.

Our applied PAR-CLIP-Array method thus identified association of only 1/3^rd^ of present miRNAs to all Argonaute proteins, while the majority of miRNAs and consequently mRNAs were specifically associated with only one or two Argonaute proteins in a cell-context specific manner.

### According to bioinformatic predictions Argonaute-miRNA complexes may regulate AML-relevant pathways in concerted action

More than one third of human genes are predicted to be under miRNA control [27]. 16.2% and 42.7% of detected mRNAs of the total RNA of KASUMI-1 and NB4 cells, respectively, were regulated by at least one Argonaute protein according to our data. Hence, the overall extend of miRNA-mediated regulation seemed to be higher in the more differentiated NB4 cell line. We identified putative miRNA targets associated with human Argonaute proteins in KASUMI-1 and NB4 cells using different target prediction algorithms (TargetScan, PicTar and miRanda) (Tables S2, S3). In KASUMI-1 cells, we found binding sites for 98.4% of Ago-associated miRNAs on 65.8% of Ago-associated mRNAs. Correspondingly, in NB4 cells, 77.3% of Ago-associated mRNAs were predicted to offer binding sites for 91.5% of Ago-associated miRNAs. miRNAs or mRNAs for which no binding site could be predicted might be regulated by a different mechanism than incorporated in the prediction algorithms like miRNA binding to the 5′-untranslated region or in the coding sequence.

**Figure 4:**
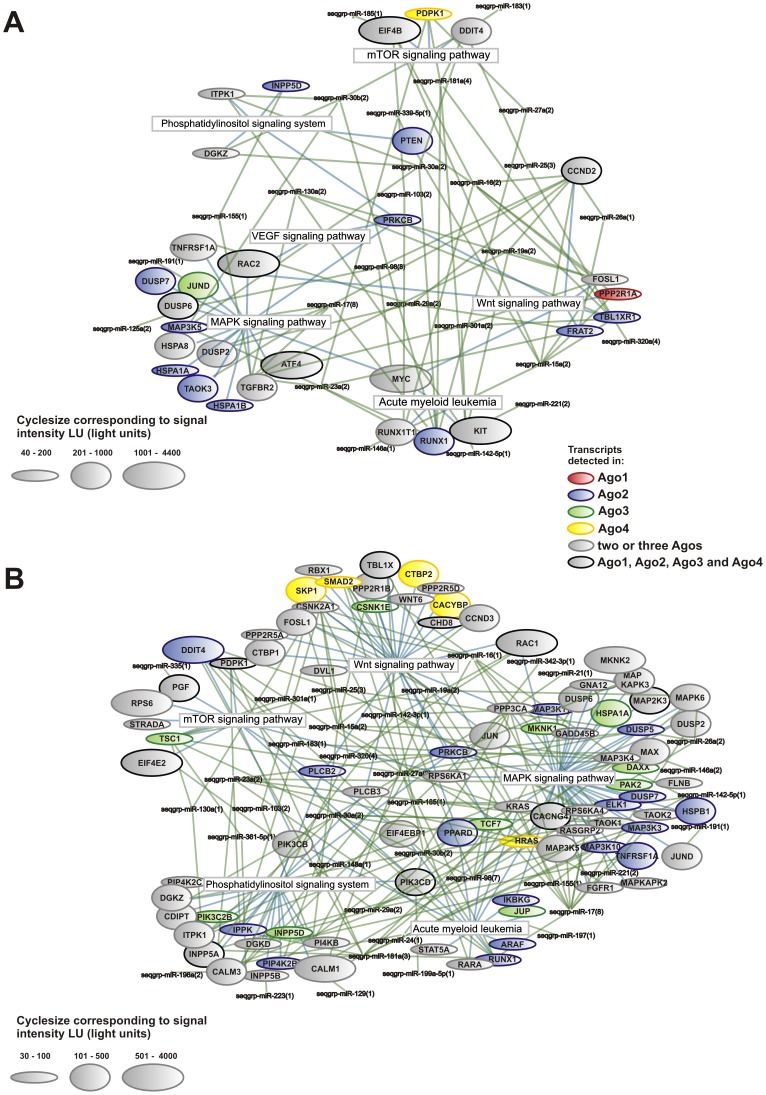
Network visualization of AML-relevant pathways enriched in the four human Argonaute proteins. Ago-protein associated mRNAs (bubbles) were mapped on KEGG signaling pathways (blue edges) in (A) KASUMI-1 and in (B) NB4 cell lines. Depicted mRNAs are enriched in the Ago-protein compared to the isotype control in triplicate experiments and were among the top enriched pathways. Bubble sizes illustrate the amount of detected mRNAs as signal intensities in light units (LU). The bubble-colors represent the different Ago-proteins in which the distinct mRNA was identified. miRNA binding sites were identified using TargetScan, PicTar and miRanda (green edges). miRNAs with sequence similarity are summarized in sequence groups (seqgrp).

In order to investigate the interplay between miRNAs and their mRNA targets, we performed KEGG pathway analyses. Overall, 93 and 118 different KEGG pathways could be identified in KASUMI-1 and NB4 cells, respectively. Nearly half of these pathways (46.2% and 48.3%) were associated with all four human Argonaute proteins in those cell lines. In contrast, only 8% and 12% of Ago-associated mRNAs could be identified in all four Argonaute proteins of KASUMI-1 and NB4 cells, respectively, suggesting a concerted action of the four Argonaute proteins in post-transcriptional mRNA regulation primary on pathway level (Figure 4; Figure S4).

mRNAs involved in ribosome, phagosome, lysosome, spliceosome, cell cycle and metabolic pathways showed high enrichment over all probed transcripts in association with the four Argonaute proteins, whereas the mTOR, MAPK, Phosphatidylinositol and Wnt signaling pathways were detected in association with at least two Argonaute proteins (Tables S2, S3). These signaling pathways showed highest enrichment or highest signal intensity in at least one Ago complex of the two AML cell lines, but not in an unrelated glioblastoma cell line (Table 3). Further analysis of the AML, MAPK and mTOR signaling pathways affirmed the collaborative function of different Ago proteins in regulation of these important pathways (Figure S4). Counter intuitively, in NB4 cells both, inhibitors like TSC1 and activators like PDPK1, were found in Argonaute complexes with multiple binding sites to co-precipitated miRNAs. However, with regard to the miRNA molecular levels bound to Argonaute complexes as quantified by our miRNA microarray approach, TSC1 was down regulated by twice the molecular amount of miRNAs as PDPK1. Given the one-to-one molecular interaction of miRNA and its mRNA target within the targeting complex, the down regulation results in a potential overall relieve of inhibition and subsequent activation of the mTOR-signaling pathway.

**Table 3:** Pathways with highest enrichment and highest signal intensity of the four human Argonaute proteins in AML cell lines KASUMI-1 and NB4.

	Pathways with highest enrichment			Pathways with highest signal intensity		
Pathway Name	KASUMI-1	NB4	SNB19	KASUMI-1	NB4	SNB19
**Acute myeloid leukemia**				Ago1, 2		
**Adherens junction**				Ago1	Ago1, 2, 4	Ago2, 3
**Aminoacyl-tRNA biosynthesis**	Ago2**			Ago2	Ago2	
**Antigen processing and presenta.**					Ago1, 3	
**Axon guidance**					Ago2, 3	
**B cell receptor signaling pathway**	Ago1***					
**Base excision repair**		Ago1**		Ago2	Ago1, 3	
**Cell cycle**	Ago1**, 2***	Ago1-4***	Ago1-3***			
**Cysteine and methionine meta.**			Ago1***	Ago3, 4		
**Drug metabolism - other enzymes**				Ago3		
**Endocytosis**	Ago2***	Ago2***, 3***				
**Fc epsilon RI signaling pathway**	Ago2***					
**Fc gamma R-mediated phagocyt.**	Ago2***				Ago4	
**Fructose and mannose metabolism**					Ago4	Ago2, 3
**Glutathione metabolism**	Ago1***, 3**, 4*					
**Glycolysis/Gluconeogenesis**				Ago1-4	Ago2	Ago2, 3
**Glyoxylate and dicarboxylate meta.**				Ago2		Ago1
**Hematopoietic cell lineage**				Ago2	Ago4	
**Leukocyte transendothelial migra.**					Ago2, 4	
**Long-term potentiation**				Ago3, 4		
**Lysosome**	Ago1***, 2***	Ago3***, 4***	Ago1-3***			
**MAPK signaling pathway**	Ago2***, 3**	Ago2***, 3***, 4***				
**mTOR signaling pathway**	Ago4**	Ago1***			Ago1, 3, 4	
**N-Glycan biosynthesis**					Ago1, 2	Ago1
**NOD-like receptor signaling pathw.**					Ago1, 3	
**Oxidative phosphorylation**	Ago1-4***	Ago2-4***	Ago1-3***	Ago2		Ago1, 3
**Pentose phosphate pathway**						Ago2, 3
**Phagosome**	Ago1***,3***, 4*	Ago2***, 3***	Ago1-3***	Ago1, 4	Ago1, 2, 4	Ago2, 3
**Phosphatidylinositol signaling sys.**		Ago2***, 3***				
**PPAR signaling pathway**	Ago2**, 4**					
**Propanoate metabolism**				Ago1-4		
**Proteasome**	Ago1***,3***, 4*		Ago1*,2-3***		Ago2	Ago1
**Protein export**	Ago4*					Ago1, 3
**Purine metabolism**	Ago3**	Ago2***, 4***	Ago1**, 2-3***			
**Pyrimidine metabolism**		Ago2***	Ago1**, 2-3***			
**Pyruvate metabolism**				Ago1-4		Ago1
**Regulation of actin cytoskeleton**					Ago2, 4	Ago2, 3
**RNA degradation**	Ago1***	Ago4***				
**RNA polymerase**		Ago2***	Ago2**, 3***			
**SNARE interactions in vesi. trans.**				Ago1		
**Spliceosome**	Ago1-4***	Ago3***, 4***	Ago1***, 3***			
**Steroid biosynthesis**	Ago3**					
**Tight junction**					Ago2, 4	Ago2
**Toll-like receptor signaling pathway**		Ago1***				
**Ubiquitin mediated proteolysis**	Ago1**	Ago2-4***	Ago1***, 3***	Ago3, 4		
**Valine, leucine and isoleucine deg.**					Ago1, 3	
**Vasopressin-regulated water reab.**	Ago4*			Ago2-4		
**VEGF signaling pathway**				Ago1		
**Wnt signaling pathway**		Ago1**				

Pathways were also compared to data from unrelated astrocytoma cell line SNB19 to determine AML specific pathways associated with miRNA regulatory machinery. Significance levels: *p*<0.05 = *; *p*<0.01 = **; *p*<0.001 = **.

In contrast to the specific binding pattern of single miRNAs and mRNAs, bioinformatics target prediction and pathway analysis based on targeting-complex- associated molecules revealed that nearly half of all detected pathways were associated with all four Argonaute proteins. Of note pathways, previously identified to be of importance for acute myeloid leukemias, were enriched in this analysis suggesting a concerted regulatory action of those pathways in our cell line models.

## Discussion

miRNAs, small regulatory non-coding RNAs, have been shown to be important regulators for various cellular processes including apoptosis and cell differentiation. Therefore, it is not astonishing, that they are not only implicated in solid malignancies, but also in hematological malignancies as adulthood AML [2]. One pediatric study with 50 AML cases of two different cytogenetic subtypes also reported that miRNAs can distinguish between morphological FAB subtype M1-3 [3]. Since more elaborate studies in pediatric AML with other cytogenetic subtypes including MLL-rearranged types are lacking so far and pediatric AML has distinct cytogenetic and clinical features compared to their adult counterparts, we performed expression profiling of 102 pediatric AML samples with distinct cytogenetic subtypes (Table 1) using a quantitative miRNA microarray approach. Four major patient groups were identified by unsupervised hierarchical clustering based upon the miRNA expression. Samples carrying the translocations t(8;21) and t(15;17) clustered together within each group, but separated from each other. A separate clustering of the core-binding factor AML, encompassing the t(8;21) and inv(16) samples, has been described in adult patients [11], [12]. In our pediatric patient cohort samples with inv(16) also clustered together with t(8;21), however, a clear separation from other cytogenetic aberrations could not be observed for inv(16), since other samples with inv(16) were also interspersed in the other clusters. No clustering pattern could be observed specific for the majority of different MLL- rearranged pediatric AML samples in our cohort, albeit class prediction analysis could predict MLL- rearranged patient samples in 30 out of 33 cases. Recently, three out of four selected miRNAs in MLL-rearranged samples are being reported as differentially expressed in a pediatric MLL-rearranged cohort [4]. In this study, we could also identify miR-196b as being expressed in a majority of MLL-rearranged samples. For miR-196a and miR-29a, both reported to be differentially expressed, no consistent change in miRNA level could be detected in our patient cohort by quantitative microarray approach. We noticed no overlap in miRNA change in the 33 MLL-rearranged samples from our study compared to nine adulthood MLL-rearranged AML samples investigated by Garzon et al. [39]. However, miRNAs found to be significantly down regulated in our cohort (miR-126-3p, miR-146a, miR-181a/b/d) were also found in a larger adulthood study of 30 MLL-rearranged patient samples [12]. Other miRNAs described by Li *et al.* (miR-130a, miR-181c and miR-224) were also down regulated in our cohort, but not at significant levels. Still other miRNAs are differentially expressed in our pediatric MLL cohort, but not in adulthood MLL samples (Table 2). Most noticeable, miR-21, a known oncomir, was over nine fold up regulated in MLL-rearranged pediatric patient samples compared to the other samples and has not been described in this context in adulthood MLL-rearranged samples.

Only a few miRNAs distinguished the t(8;21) and t(15;17) subtypes from each other and all other cytogenetic subtypes, which is coherent with previous reports of miRNA expression in adulthood AML [12], [40]. Some observed differences of miRNA expression between patients with t(8;21) and t(15;17) seemed to be a function of maturation state of the cell as is the case for miR-23b, that is up regulated during hematopoietic differentiation from CD34+ cells to more mature peripheral blood cells [40]. Similarly, we identified miR-181a/b higher expressed in t(15;17) than in other cytogenetic subtypes and normal hematopoietic progenitor cells as has been reported for adult myeloid AML cells in comparison to monocytic AML [40], [41]. The miRNA let-7a has been reported to be up regulated upon differentiation of the promyelocytic cell line NB4 [42] and in our study this miRNA was low expressed in this cell line as well. However, in initial untreated samples of pediatric APL patient samples it was more than 2-fold higher expressed. Other differentially expressed miRNAs have already been described in the context of myeloid malignancy development including miR-100, miR-125b, miR-126, miR-146a, miR-150 and miR-181a/b. We identified miR-100 and miR-125b up regulated in pediatric acute promyelocytic leukemia consistent with results from a smaller cohort of pediatric AML patients [3] and from adult patients [12]. Henson et al. demonstrated, that miR-100 and miR-125b significantly decrease cell proliferation [43]. Consistently, miR-125b overexpression leads to malignant transformation of different hematopoietic lineages in a mouse model [44], [45]. Similarly, miR-126 is significantly higher expressed in pediatric samples with t(8;21) than in other pediatric subgroups in concordance with previous published adulthood studies [12]. This miRNA was shown to inhibit apoptosis thus increasing viability in AML cell lines by the same study and was selected as one of the class identifier miRNAs in our study. We identified miR-146a, whose inhibition induces myeloid malignancies in a mouse model [46], as higher expressed in both translocations belonging to the good prognosis group in comparison to the other translocations belonging to more high risk groups in our study. Although miR-223 activation is induced and important in myelocyte differentiation into granulocytes [47]–[49], we could detect higher levels in the t(8;21) cohort bearing the AML1/ETO fusion protein compared to more mature promyelocytic leukemias. Similar observations have been made for adulthood M1/M2 leukemia compared to M3 AML [50]. This is in contrast to the low expression of miR-223 reported in four patients bearing a t(8;21) compared to other patients by Fazi *et al.* [51]. Differences in the three studies might be due to the patient cohort related to low patient numbers in the report by Fazi *et al.* However, the low expression of miR-223 is rather unexpected because the AML1/ETO fusion protein is thought to inhibit miR-223 expression by recruiting chromatin remodeling enzymes [51] and expression of this miRNA was lower in the KASUMI-1 cell line bearing the translocation than in other cell lines. Secondly, miR-223 expression is thought to be driven by transcription factor C/EBPα [48] and this transcription factor is about ten-fold higher expressed in t(15;17) adulthood patients than in t(8;21) patients [52]. A possible explanation is alternative usage of other transcription factors like C/EBPβ and PU.1 as demonstrated for mouse miR-223 promoter [53].

Comparing the miRNA profiles of our pediatric cohort to adult AML samples carrying t(8;21) and t(15;17) we noticed that five out of six adult AML patient samples clustered together irrespectively of translocation. Age-related miRNA expression changes and their role in longevity has been described in *C.elegans* [54]. In human peripheral mononuclear cells Noren Hooten et al. described an age-specific down regulation of miRNA expression in adult compared to pediatric samples [55]. However, out of the nine miRNAs significantly lower expressed in their adult cohort compared to the pediatric one, only miR-221 was found to be 2.2- and 2.6-fold higher expressed in children compared to their adulthood counterpart in this study for t(8;21) and t(15;17)-positive patient samples, respectively. Influence of age on overall survival for those two cytogenetic subgroups has been reported previously, but not for patients under the age of 49 years [56]. The age range for the three t(8;21)-positive adulthood patients was 34–50 years. Therefore it is unlikely, that observed differences between pediatric and adulthood AML samples with the same cytogenetic aberration is due to age-related effects. The influence of differences in other molecular characteristics, such as observed more frequent activating mutations in *ckit* in adult than in pediatric core-binding factor AML [57], on miRNA expression was beyond the scope of this work and has to be addressed by further detailed studies. However, we find the ckit mRNA associated with all four Ago proteins together with miR-18b, which is lower expressed in t(8;21)- positive leukemia in our pediatric cohort relative to other cytogenetic subtypes but also relative to the examined adulthood t(8;21) patients (∼70-fold, p = 0.003). On the basis of our data presented here we hypothesize, that activating mutations of *ckit* makes its deregulation by miRNAs dispensable for the CBF-AML in adults but not in children, however, this is awaiting future functional studies.

Since it is known that miRNAs bind to mRNAs within the Argonaute proteins for post-transcriptional gene silencing, we isolated the four different Argonaute protein complexes to determine the bound miRNAs and mRNAs. This was much needed, because different bioinformatic target predictions use upfront for a given miRNA yield thousands of targets and only overlap by ∼30%. Proteomics data measuring protein level changes after overexpression or down regulation of a single miRNA reveal a false-positive rate for bioinformatic target predictions of at least 34% [58], [59]. We established a modified PAR-CLIP method [26] called PAR-CLIP-Array for Argonaute complex isolation in two cell lines, KASUMI-1 and NB4, carrying the translocation t(8;21) or t(15;17), that were the most distinctive AML subtypes identified in our pediatric patient cohort. In our method, we combined for the first time previously successful Argonaute isolation methods (Figure 2). Although monoclonal antibodies for Argonaute precipitation followed by microarray detection of bound miRNAs and mRNAs have been used before [22], we used photo-activatable cross-linking in order to avoid re-association of RNA sequences to RNA-binding proteins after cell lysis [38]. Monoclonal antibodies were used with an isotype antibody control rather than overexpression of tagged proteins since this might again introduce a shifting of bound miRNAs and mRNAs. In fact, we detected bound mRNAs to the isotype control, despite stringent washing conditions, although no Argonaute protein could be detected on Western blot analysis. A further increase of washing conditions resulted in loss of Argonaute protein (data not shown). This further emphasizes the need for the use of isotype controls in immunoprecipitation experiments. Unsupervised hierarchical clustering of Ago-associated miRNAs and mRNAs revealed distinct binding preferences for both molecules of Argonaute proteins in both cell lines, since the t(8;21)- and t(15;17)-positive cell lines clustered more separately from each other than total RNA from both cell lines (Figure S2). Together with the expression data in our pediatric patient cohort, this further underscores the differences between those two subtypes not only in miRNA levels, but consecutively also in mRNA targeting. To our surprise given the structural similarity of the Argonaute proteins and previous reports of similar binding of mRNAs to Argonaute proteins using a tagged protein overexpression approach in HEK293 cell line [60] and miRNAs using monoclonal antibodies in THP1 cell line [25] and in mouse skin [20], also the human Argonaute proteins clustered completely separately with only 8% and 12% of associated mRNAs overlapping between all four Ago proteins in KASUMI-1 and NB4 cell lines, respectively. This is consistent with mRNA alterations after knock-down of individual Ago proteins in the HEK293 cell line [61], although perturbing the Ago expression could also result in other bystander effects. Although differential sorting of siRNAs and miRNAs in fly and worm is appreciated for some time and several determinants have been identified [62], [63], our study directly identified differential and exclusive sorting of miRNAs to Argonaute proteins dependent on cell context for the first time so far. The differences in associated miRNAs and mRNAs in a cell context-specific manner might result in differential mode of miRNA function especially for association to Ago2, since this Argonaute protein is the only one in humans possessing slicing activity [64], [65], undergoes nuclear-cytoplasmatic shutteling [66] and has recently been implicated in transcriptional gene silencing in senescence [67]. In our study, we exclusively identified 46 miRNAs in association with Ago2 in the KASUMI-1 cell line including members of the let-7 family of miRNAs (let-7b/c/d/e/f/i), that has been implicated in transcriptional gene silencing. Five miRNAs (miR-185, 196a/b, 422a and 423) could be exclusively identified in association with Ago2 in both, the KASUMI1 and NB4 cell lines. In NB4 cells nearly 73% of Ago2 and Ago3-associated miRNAs could be identified in both Argonaute proteins. The binding of largely overlapping subsets of miRNAs to human Ago2 and Ago3 has been reported previously in the T-lymphocytic Jurkat cell line [18]. Although each Argonaute can rescue the cell death phenotype of mouse embryonic stem cells [68], it has been demonstrated that miR-24 and miR-199a-3p is reduced in respective mAgo2−/− mouse embryonic fibroblasts, but not in mAgo1−/− and mAgo3−/− animals [69]. In the KASUMI-1 cell line we also identified miR-24 as 8-fold more associated with Ago2 (p = 0.037, two-sample t-test for unequal variances) and miR-199a-3p mainly associated with Ago2, whereas in the more differentiated promyelocytic cell line NB4 miR-24 and miR-199a-3p were associated with Ago1-3.

Mapping the associated mRNAs with miRNA binding sites on KEGG pathways illustrated a concerted action of all four Ago proteins in regulation of about half of KEGG-annotated pathways. This is consistent with the observed overall functional redundancy of Ago proteins in Ago1-4 deficient mouse embryonic stem cells in rescuing the apoptosis phenotype by reintroduction of any Ago protein [68]. Among the top enriched and highly expressed Argonaute protein associated pathways are MAPK [70], mTOR, Phosphatidylinositol [71] and Wnt [72] pathways (Figure 4; Figure S4), that have been described as deregulated and important for the survival of AML cells. In KASUMI-1 cell line it is possible that the classical MAP-kinase signaling pathway is activated due to the repression of DUSP phosphatase by four different miRNA sequence groups (seqgrp-miR-29a, 17, 98 and 125a) we identified in association with all four Argonaute proteins. This could lead to stimulation of proliferative genes by activation of the mitogen-activated protein kinase ERK and to uncontrolled cell growth and survival of leukemic cells. Indeed, these relations have to be proved in further investigations.

An up regulation of the *mir-23-27-24* gene clusters by binding of the AML1/ETO fusion protein to its promoter was described in KASUMI-1 cell line [70]. DUSP16 (also designated as MKP-7) was identified as a target using the erythroleukemia cell line K562 upon overexpression of *mir-24*. However, DUSP16 was not identified in Ago complexes in the KASUMI-1 cell line by our study, indicating that it may not be a true target in the KASUMI-1 cell context. We identified two other members of the MAP kinase pathway to be regulated by these miRNA clusters as complexed to the Ago2 and Ago3 proteins. TGFß-receptor 2 and downstream apoptosis signal regulated kinase 1 (syn. MAP3K5) of the p38 MAP kinase pathway are both complexed together and have a binding site for miR-23a/b, but not for the other two members of the primary transcript, miR-24 and miR-27.

Overall, we extended previous profiling studies in pediatric AML identifying four different subgroups based upon miRNA expression and could identify previously described deregulated miRNAs between t(8;21)- and t(15;17)-positive samples as well as previously not recognized differentially expressed miRNAs. Moreover, we experimentally identified miRNAs and mRNAs associated with the Argonaute complexes using a modified PAR-CLIP-Array method. This is the first time, this technique has been applied to AML cells and provides insights into the complexity of regulation of AML-relevant pathways by concerted action of different Ago proteins. In addition, this work gives the scientific community a reliable experimental resource for future functional testing of single miRNA-mRNA function in core-binding factor AML and promyelocytic AML, since we avoided artifacts by using native cell lines that do not over express tagged Argonaute proteins.

## Supporting Information

Figure S1 miRNA predictive signature for t(8;21), t(15;17) and MLL-rearranged patient samples. The PAM algorithm selected significantly regulated miRNAs as class identifiers from patient samples with (A) all cytogenetic subtypes or with (B) t(8;21)-, t(15;17)- and MLL-rearranged subtypes. The centroid plots for each miRNA is given as well as the predictive statistics from 10-fold cross-validation. Se, Sensitivity as positively predicted vs. true positive for each class; Sp, Specificity as negatively predicted vs. true negative for each class. Please note that no sample belonging to the inv(16), normal karyotype or other are correctly predicted and thus the specificity and overall accuracy was not calculated (n/a, not applicable). Fold changes are indicated in Table 2.
(TIF)

Figure S2 Ago-associated miRNAs and mRNAs of KASUMI-1 and NB4 cell lines. (A) miRNAs and (B) mRNAs associated to Ago-proteins, to the isotype control antibody and in total RNA were analyzed by microarray technology as given in detail in “Materials and Methods”. Unsupervised hierarchical clustering based upon these expression profiles were generated on the complete data set, while the heatmaps show filtered data of (A) miRNAs and (B) mRNAs differentially associated with the Ago-proteins of a given cell line with a significance level of p<0.05 in Student's t-testing and a fold change of >1.8-fold.
(TIF)

Figure S3 Partial overlap of Ago-associated miRNAs and mRNAs of KASUMI-1 and NB4 cells. (A) Relationship between Ago-associated miRNAs and miRNAs identified in total RNA in the KASUMI-1 (left) and the NB4 cell line (right). (B) Relationship between Ago-associated mRNAs and mRNAs identified in total RNA in the KASUMI-1 (left) and NB4 cell line (right). The total number of RNAs is given at the outer border of each set (n = x).
(TIF)

Figure S4 Ago-associated miRNA regulatory network in selected AML-relevant pathways. Ago-associated mRNAs and miRNAs were mapped upon pathways from the KEGG database. The drawing was adapted from the KEGG database. Boxes delineate gene products of mRNAs identified from Ago-complexes in KASUMI-1 (red), NB4 (yellow) or both (red/yellow) cell lines. Next to the gene products little squares indicate the amount of mRNAs associated with the Ago-proteins and identified in total RNA (T) in the respective color as indicated in the figure. miRNAs with sequence similarity were summarized in sequence groups (seqgrp) or families and miRNAs with binding sites to the respective mRNA are given. Sequence group names are preceded by a dot (•) if miRNAs were identified as differentially expressed between t(8;21)- samples and t(15;17) samples compared to all others in our pediatric AML patients cohort.
(TIF)

Table S1 Comparison of miRNA expression between pediatric patient sample and respective cell lines with t(8;21) and t(15;17). Abbreviations: SI,-signal intensity (sample/UR); UR,-universal reference; n.d., not detected.
(XLS)

Table S2 Identification of KEGG pathways associated with Ago-protein bound mRNAs and miRNAs in KASUMI-1 cell line. mRNAs associated to Ago1, 2, 3, and 4 as well as mRNAs identified in total RNA are given with identified KEGG pathways in each spreadsheet. miRNA binding sites identified by at least two of the three used algorithms (TargetScan, PicTar, MiRanda) are given.
(XLS)

Table S3 Identification of KEGG pathways associated with Ago-protein bound mRNAs and miRNAs in NB4 cell line. See also caption Table S2.
(XLS)

Table S4 Ago-associated mRNAs of KASUMI-1 and NB4 cell lines referring to Figure S2B. Gene list of the corresponding mRNAs associated with the four human Argonaute proteins of KASUMI-1 and NB4. The ordering of this gene list corresponds to the ordering of the hierarchical clustering shown in Figure S2B. The levels of hierarchical clustering as well as the functional gene groups are given. Significantly enriched GO terms and KEGG pathways representative of the gene group are indicated.
(XLS)
