# Supplementary figures and images for: MicroRNAs Distinguish Cytogenetic Subgroups in Pediatric AML and Contribute to Complex Regulatory Networks in AML-Relevant Pathways
Source: PLoS One. 2013 Feb 13;8(2):e56334. doi: 10.1371/journal.pone.0056334 (PMC3572007; doi:10.1371/journal.pone.0056334)

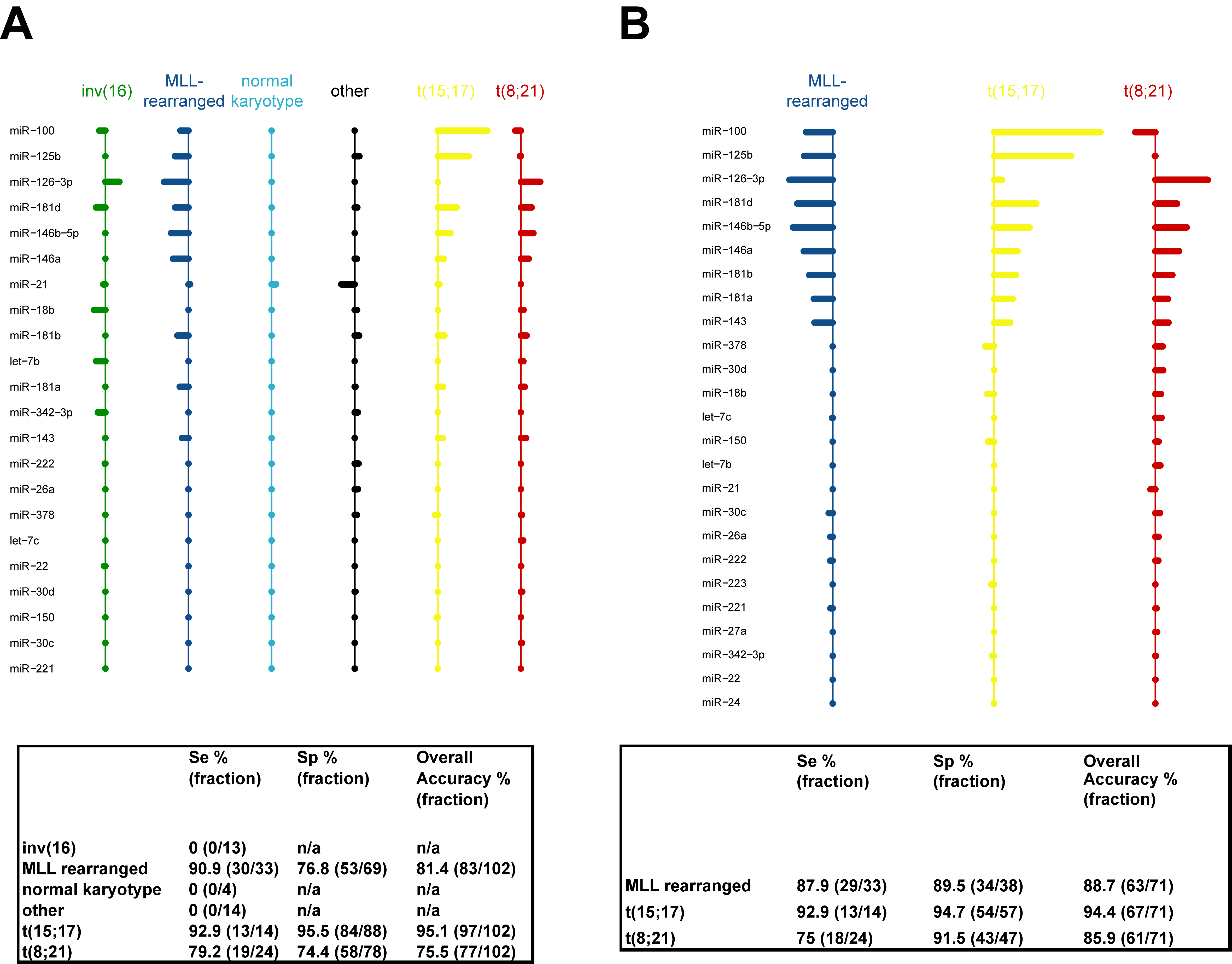

Supplement: Figure S1 — miRNA predictive signature for t(8;21), t(15;17) and MLL-rearranged patient samples. The PAM algorithm selected significantly regulated miRNAs as class identifiers from patient samples with (A) all cytogenetic subtypes or with (B) t(8;21)-, t(15;17)- and MLL-rearranged subtypes. The centroid plots for each miRNA is given as well as the predictive statistics from 10-fold cross-validation. Se, Sensitivity as positively predicted vs. true positive for each class; Sp, Specificity as negatively predicted vs. true negative for each class. Please note that no sample belonging to the inv(16), normal karyotype or other are correctly predicted and thus the specificity and overall accuracy was not calculated (n/a, not applicable). Fold changes are indicated in Table 2. (TIF) [file pone.0056334.s001.tif]

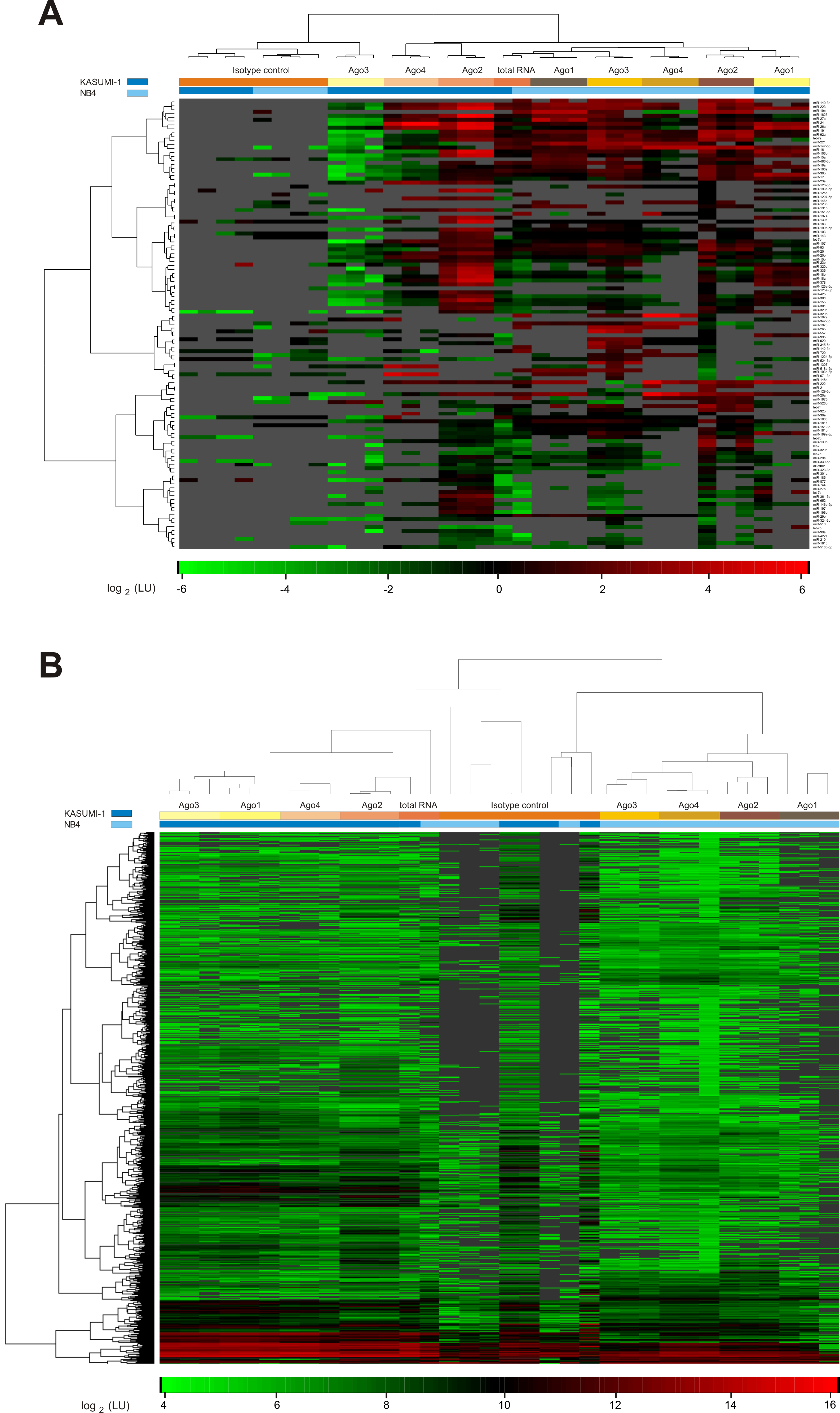

Supplement: Figure S2 — Ago-associated miRNAs and mRNAs of KASUMI-1 and NB4 cell lines. (A) miRNAs and (B) mRNAs associated to Ago-proteins, to the isotype control antibody and in total RNA were analyzed by microarray technology as given in detail in “Materials and Methods”. Unsupervised hierarchical clustering based upon these expression profiles were generated on the complete data set, while the heatmaps show filtered data of (A) miRNAs and (B) mRNAs differentially associated with the Ago-proteins of a given cell line with a significance level of p<0.05 in Student's t-testing and a fold change of >1.8-fold. (TIF) [file pone.0056334.s002.tif]

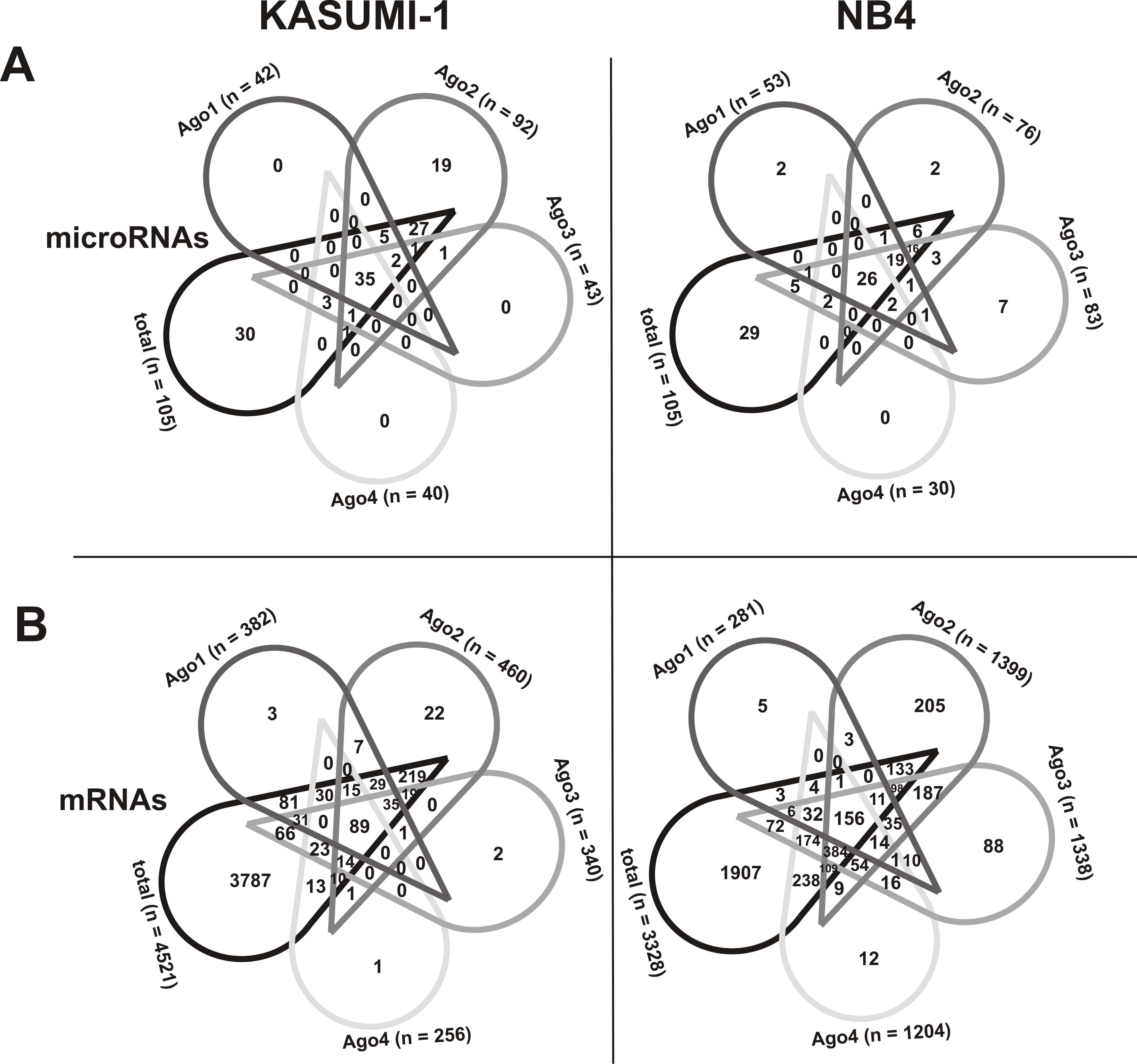

Supplement: Figure S3 — Partial overlap of Ago-associated miRNAs and mRNAs of KASUMI-1 and NB4 cells. (A) Relationship between Ago-associated miRNAs and miRNAs identified in total RNA in the KASUMI-1 (left) and the NB4 cell line (right). (B) Relationship between Ago-associated mRNAs and mRNAs identified in total RNA in the KASUMI-1 (left) and NB4 cell line (right). The total number of RNAs is given at the outer border of each set (n = x). (TIF) [file pone.0056334.s003.tif]

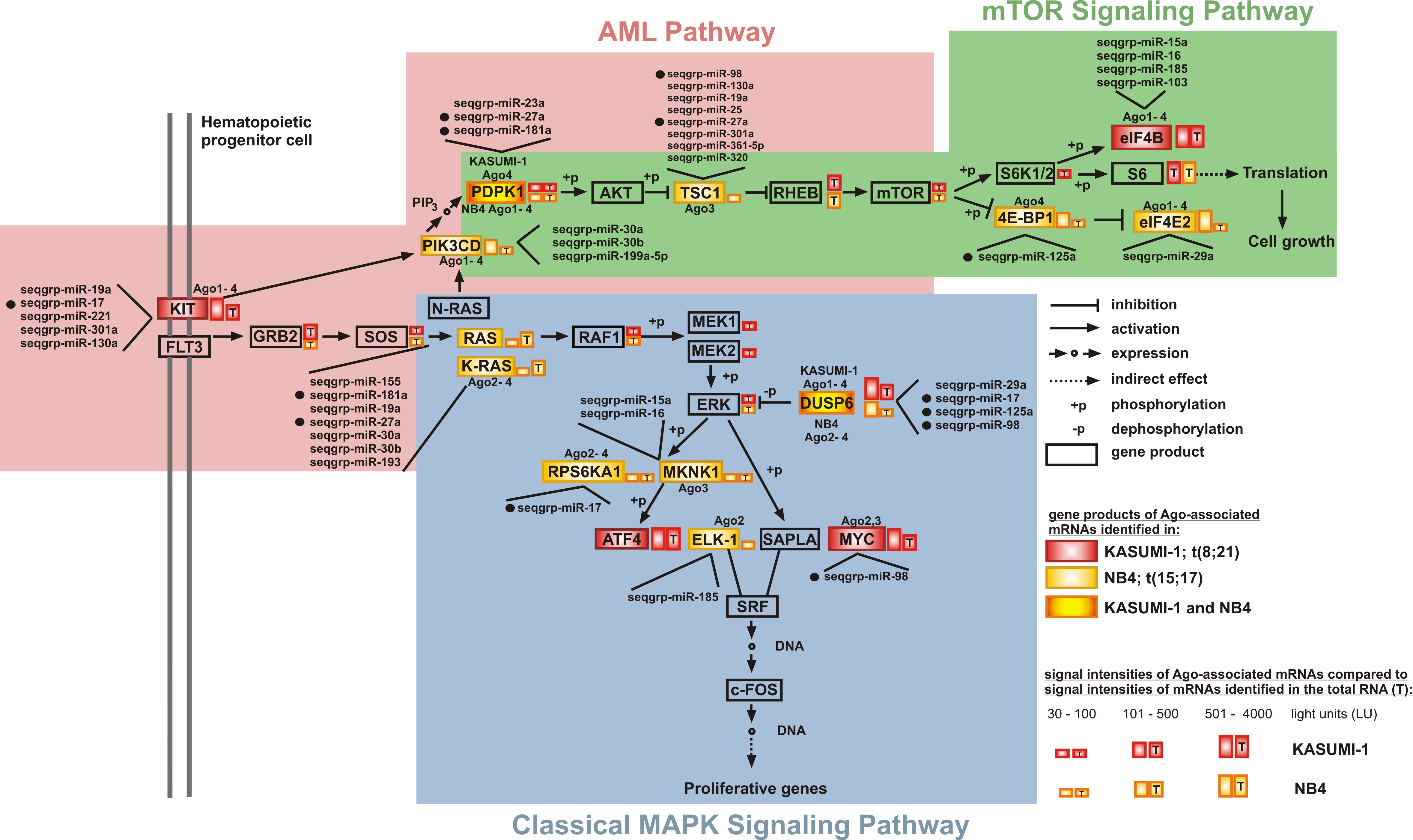

Supplement: Figure S4 — Ago-associated miRNA regulatory network in selected AML-relevant pathways. Ago-associated mRNAs and miRNAs were mapped upon pathways from the KEGG database. The drawing was adapted from the KEGG database. Boxes delineate gene products of mRNAs identified from Ago-complexes in KASUMI-1 (red), NB4 (yellow) or both (red/yellow) cell lines. Next to the gene products little squares indicate the amount of mRNAs associated with the Ago-proteins and identified in total RNA (T) in the respective color as indicated in the figure. miRNAs with sequence similarity were summarized in sequence groups (seqgrp) or families and miRNAs with binding sites to the respective mRNA are given. Sequence group names are preceded by a dot (•) if miRNAs were identified as differentially expressed between t(8;21)- samples and t(15;17) samples compared to all others in our pediatric AML patients cohort. (TIF) [file pone.0056334.s004.tif]
